# Correction: Yaseen et al. Photo-Assisted Removal of Rhodamine B and Nile Blue Dyes from Water Using CuO–SiO_2_ Composite. *Molecules* 2022, *27*, 5343

**DOI:** 10.3390/molecules29235732

**Published:** 2024-12-05

**Authors:** Muhammad Yaseen, Muhammad Humayun, Abbas Khan, Muhammad Idrees, Nasrullah Shah, Shaista Bibi

**Affiliations:** 1 Department of Chemistry, Abdul Wali Khan University, Mardan 23200, Pakistan; 2 Wuhan National Laboratory for Optoelectronics, School of Optical and Electronic Information, Huazhong University of Science and Technology, Wuhan 430074, China; 3 Additive Manufacturing Institute, College of Mechatronics and Control Engineering, Shenzhen University, Shenzhen 518060, China

## Missing Citation

Citation of the publication (our own previous work), Yaseen, M.; Farooq, S.; Khan, A.; Shah, N.; Shah, L.A.; Bibi, S.; Khan, I.U.; Ahmad, S. CuO-SiO_2_ based nanocomposites: Synthesis, characterization, photocatalytic, antileishmanial, and antioxidant studies. *J. Chin. Chem. Soc.* **2022**, *69*, 1637–1653 was not cited in the figure captions of Figures 1–4 of the original article [1].

The citation has now been inserted in Section 2, Figures 1–4:

**Figure 1.** (**a**) UV−Visible spectrum; (**b**) Taucs plot for bandgap calculation; and (**c**) FT-IR spectrum of CuO–SiO_2_ composite. Reproduced with permission from JCCS, Wiley, 2022 [23].

**Figure 2.** (**a**) SEM and (**b**) TEM images; and (**c**) EDX spectra of CuO–SiO_2_ composite. Reproduced with permission from JCCS, Wiley, 2022 [23].

**Figure 3.** (**a**) XRD spectra of CuO particles and CuO–SiO_2_ composite; and (**b**) TGA curve of CuO–SiO_2_ composite. Reproduced with permission from JCCS, Wiley, 2022 [23].

**Figure 4.** Nitrogen adsorption isotherms for CuO-SiO_2_ composite: (**a**) isotherm linear plot; (**b**) BET surface area plot; (**c**) t-plot; and (**d**) BJH adsorption plot. Reproduced with permission from JCCS, Wiley, 2022 [23].

The authors have obtained copyright permission from the publisher of JCCS, Wiley for re-publishing the figures. The PDF version of the copyright permission is available upon request.

The authors state that the scientific conclusions are unaffected. This correction was approved by the Academic Editor. The original publication has also been updated.
