# Supplementary material for: Photo-Assisted Removal of Rhodamine B and Nile Blue Dyes from Water Using CuO–SiO2 Composite
Source: Molecules. 2022 Aug 22;27(16):5343. doi: 10.3390/molecules27165343 (PMC9413644; doi:10.3390/molecules27165343)
Supplement: Supplementary file 1 [file molecules-27-05343-s001.zip › molecules-1848280-supplementary.pdf]

Supplementary Information (SI)

Phtoto-Assisted Removal of Rhodamine B and Nile Blue Dyes from Water using $CuO-SiO_2$ Composite

Muhammad Yaseen[1], Muhammad Humayun*[2], Abbas Khan*[1], Muhammad Idrees[3], Nasrullah Shah[1], and Shaista Bibi[1]

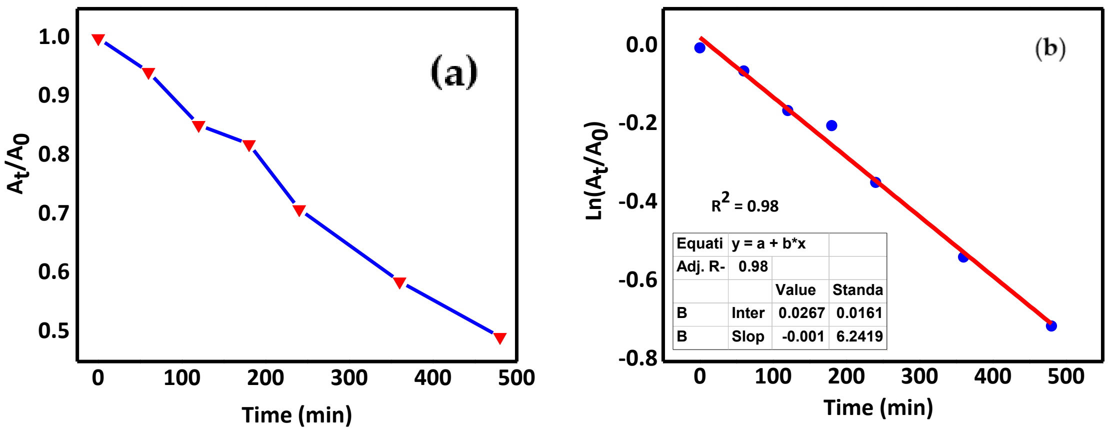

Figure S1. (a) Ratio of absorbances (A_0/A_t) versus time, and (b) characteristic plot for pseudo first order kinetics of degradation for self-degradation of RhB in the absence of $CuO-SiO_2$ particles under light

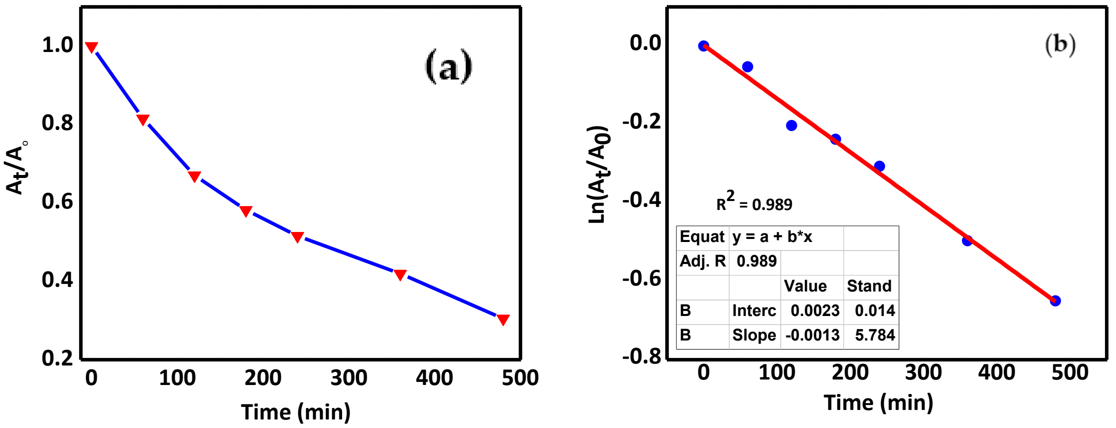

Figure S2. (a) Ratio of absorbances (A_0/A_t) versus time, and (b) characteristic plot for pseudo first order kinetics of degradation for self-degradation of NB in the absence of $CuO-SiO_2$ particles under light.
